# Protein kinase C phosphorylates AMP-activated protein kinase α1 Ser487

**DOI:** 10.1042/BCJ20160211

**Published:** 2016-12-09

**Authors:** Helen R. Heathcote, Sarah J. Mancini, Anastasiya Strembitska, Kunzah Jamal, James A. Reihill, Timothy M. Palmer, Gwyn W. Gould, Ian P. Salt

**Affiliations:** 1 Institute of Cardiovascular and Medical Sciences, College of Medical, Veterinary and Life Sciences, University of Glasgow, Glasgow G12 8QQ, U.K.; 2 Institute of Molecular, Cell and Systems Biology, College of Medical, Veterinary and Life Sciences, University of Glasgow, Glasgow G12 8QQ, U.K.

**Keywords:** AMPK, protein kinase C, vascular endothelial growth factor

## Abstract

The key metabolic regulator, AMP-activated protein kinase (AMPK), is reported to be down-regulated in metabolic disorders, but the mechanisms are poorly characterised. Recent studies have identified phosphorylation of the AMPKα1/α2 catalytic subunit isoforms at Ser487/491, respectively, as an inhibitory regulation mechanism. Vascular endothelial growth factor (VEGF) stimulates AMPK and protein kinase B (Akt) in cultured human endothelial cells. As Akt has been demonstrated to be an AMPKα1 Ser487 kinase, the effect of VEGF on inhibitory AMPK phosphorylation in cultured primary human endothelial cells was examined. Stimulation of endothelial cells with VEGF rapidly increased AMPKα1 Ser487 phosphorylation in an Akt-independent manner, without altering AMPKα2 Ser491 phosphorylation. In contrast, VEGF-stimulated AMPKα1 Ser487 phosphorylation was sensitive to inhibitors of protein kinase C (PKC) and PKC activation using phorbol esters or overexpression of PKC-stimulated AMPKα1 Ser487 phosphorylation. Purified PKC and Akt both phosphorylated AMPKα1 Ser487 *in vitro* with similar efficiency. PKC activation was associated with reduced AMPK activity, as inhibition of PKC increased AMPK activity and phorbol esters inhibited AMPK, an effect lost in cells expressing mutant AMPKα1 Ser487Ala. Consistent with a pathophysiological role for this modification, AMPKα1 Ser487 phosphorylation was inversely correlated with insulin sensitivity in human muscle. These data indicate a novel regulatory role of PKC to inhibit AMPKα1 in human cells. As PKC activation is associated with insulin resistance and obesity, PKC may underlie the reduced AMPK activity reported in response to overnutrition in insulin-resistant metabolic and vascular tissues.

## Introduction

AMP-activated protein kinase (AMPK) is a heterotrimeric Ser/Thr kinase consisting of catalytic (α) and regulatory (β and γ) subunits that acts as a key sensor of cellular and whole-body energy status [1,2]. Binding of AMP to the γ-subunit allosterically activates AMPK, promotes activating phosphorylation of AMPKα at Thr172 by the ubiquitous upstream AMPK kinase LKB1 (liver kinase B1) and inhibits Thr172 dephosphorylation, effects that are competitively inhibited by ATP [1,2]. As a consequence, AMPK is activated by conditions that increase the AMP:ATP ratio, such as hypoxia, hypoglycaemia, ischaemia and skeletal muscle contraction [1–3]. Furthermore, several pharmacological agents and xenobiotics, such as metformin, resveratrol and berberine, have been demonstrated to activate AMPK by inhibiting mitochondrial ATP synthesis and thereby increasing AMP:ATP [4]. AMPK can also be activated independent of changes in adenine nucleotide ratios by increasing intracellular Ca^2+^, in cells that express the alternative Thr172 kinase CaMKKβ (Ca^2+^/calmodulin-dependent protein kinase kinase-β) [5]. Once activated, AMPK serves to stimulate ATP synthesis and suppress ATP utilisation by multiple effects on nutrient metabolism, including the stimulation of fatty acid oxidation, muscle glucose uptake and mitochondrial biogenesis in addition to the inhibition of protein translation, fatty acid synthesis, lipogenesis and cholesterol synthesis. As a consequence, AMPK activation serves to normalise cellular adenine nucleotide ratios. Owing to these effects on nutrient metabolism, activation of AMPK has been proposed to be a therapeutic target for metabolic diseases, including diabetes and obesity [1–3]. Furthermore, AMPK has been demonstrated to have anti-inflammatory, anti-proliferative and anti-atherosclerotic actions, suggesting that it may be a useful therapeutic target in macrovascular disease, inflammatory diseases and cancer [6,7].

Despite the well-characterised mechanisms by which AMPK is activated, far less is known concerning the mechanisms that down-regulate AMPK activity, such as that reported in obese, insulin-resistant rodents and humans [8–11]. Recently, phosphorylation of AMPKα1/α2 at Ser487/491 (human sequence, equivalent to rodent Ser485/491) has been reported to inhibit AMPK activity [12–18]. Several studies have demonstrated that Akt phosphorylates AMPKα1 Ser487 in response to insulin or IGF-1 (insulin-like growth factor) in heart, adipocytes, vascular smooth muscle cells (VSMCs) and tumour cell lines [13–17]. Phosphorylation of AMPKα1 Ser487 by Akt inhibits Thr172 phosphorylation and thereby reduces AMPK activity [13,17]. Recombinant PKA (cAMP-dependent protein kinase) also phosphorylates AMPKα1 Ser487 *in vitro*, and cAMP-elevating agents have also been reported to stimulate AMPKα1 Ser487 phosphorylation in mouse embryonic fibroblasts and insulin-secreting cell lines [19,20]. Recently, inhibitors of the mitogen-activated protein kinase kinase (MEK1/2) extracellular signal-regulated kinase 1/2 (ERK1/2) and IKK (inhibitor of nuclear factor-κB kinase) pathways have also been reported to attenuate AMPKα1 Ser487 phosphorylation in human dendritic cells and a mouse macrophage cell line, implicating these pathways as regulators of AMPKα1 Ser487 [21,22].

In contrast, AMPKα2 Ser491 has been reported to be a poor substrate for Akt *in vitro* [17], although p70S6 kinase, downstream from Akt, has been reported to underlie leptin-mediated phosphorylation of AMPKα2 Ser491 in the mouse hypothalamus and a neuronal cell line [23]. Using an antibody that recognises both AMPKα1/α2 Ser487/491 phosphorylation, AMPK autophosphorylation at Ser487/491 has been reported *in vitro* [13] and the AMPK activator 5-aminoimidazole-4-carboxamide ribonucleoside (AICAR) has been reported to stimulate AMPKα1/α2 Ser487/491 phosphorylation in neonatal rat cardiomyocytes, rat VSMCs and a mouse microglial cell line [24–26].

Intriguingly, aortae from mice with experimental diabetes exhibit increased basal and IGF-1-stimulated phosphorylation of Akt and AMPKα1 Ser487, with concomitant reduced AMPKα Thr172 phosphorylation [15], and infusion of rats with glucose increased AMPKα1/α2 Ser487/491 phosphorylation [27]. Furthermore, transfection of a murine muscle cell line with AMPKα2 Ser491Ala has been recently reported to attenuate the inhibition of insulin signalling by phorbol 12-myristate 13-acetate (PMA) [18]. These studies suggest that increased AMPKα1/α2 Ser487/491 phosphorylation may underlie the reduced AMPK activity reported in insulin-resistant states [18,27]. Despite this, the AMPKα1/α2 Ser487/491 phosphorylation status in human insulin resistance has not been reported.

We have previously demonstrated that vascular endothelial growth factor (VEGF) stimulates AMPK in a CaMKK-dependent manner in human endothelial cells [28]. VEGF receptor activation in endothelial cells also stimulates Akt and ERK1/2 activity, suggesting that VEGF may be an endogenous AMPK activator that concurrently stimulates activating phosphorylation at Thr172 and inhibitory phosphorylation at Ser487. The present study aimed to examine whether VEGF promotes inhibitory AMPK phosphorylation in cultured primary human endothelial cells and define the mechanisms underlying this.

## Materials and methods

### Materials

Cryopreserved human aortic endothelial cells (HAECs), human umbilical vein endothelial cells (HUVECs) and MV2 medium were purchased from Promocell (Heidelberg, Germany). VEGF, oleoyl-2-acetyl-*sn*-glycerol (OAG) and mouse anti-FLAG tag antibodies were obtained from Sigma–Aldrich (Poole, U.K.). STO-609, CRT0066101, LY333531 and GF109203X were from Tocris (Abingdon, U.K.). A769662 and mouse anti-β-tubulin antibodies were obtained from Abcam (Cambridge, U.K.). AICAR was from Toronto Research Chemicals (Toronto, Canada). Wortmannin, PMA, Akt inhibitor VIII (Akti-1/2) and agarose-conjugated mouse anti-myc tag antibodies were obtained from Merck Millipore (Watford, U.K.). Calf intestinal alkaline phosphatase (CIAP) was from Promega (Southampton, U.K.). Opti-MEM reduced serum medium was from Life Technologies (Paisley, U.K.). HiPerFect and siRNA targeted to PKC isoforms were obtained from Qiagen (Manchester, U.K.). Rabbit anti-phospho-ACC (acetyl-CoA carboxylase; Ser79), anti-AMPKα2, anti-phospho-AMPKα (Thr172), anti-phospho-AMPKα1 (Ser485), anti-phospho-AMPKα1/α2 (Ser485/Ser491), anti-phospho-Akt (Ser473), anti-ERK1/2, anti-phospho-MARCKS (myristoylated alanine-rich PKC substrate; Ser152/Ser156), anti-protein kinase C (PKC)α, anti-PKCζ, anti-phospho-protein kinase D (PKD)/PKCµ (Ser916), anti-PKD/PKCµ antibodies and mouse anti-Akt, anti-phospho-ERK1/2 (Thr202/Tyr204) antibodies were from New England Biolabs UK (Hitchin, U.K.). Rabbit anti-PKC (pan), anti-PKCη and anti-PKCβ1 antibodies were from Santa Cruz Biotechnology, Inc. (Dallas, TX, U.S.A.). Mouse anti-PKCγ, anti-PKCδ, anti-PKCε, anti-PKCθ and anti-PKCλ antibodies were from BD Transduction Laboratories (Oxford, U.K.). IRdye680- or 800-labelled donkey anti-mouse IgG and anti-rabbit IgG antibodies were from LiCor Biosciences (Lincoln, U.K.). Lipofectamine 2000, Medium 199, mouse anti-GAPDH (glyceraldehyde 3-phosphate dehydrogenase) and Alexa Fluor 680 donkey anti-sheep IgG antibodies were from Life Technologies (Paisley, U.K.). Purified rat brain PKC was obtained from Promega (Manchester, U.K.). Purified human recombinant Akt1 was obtained from Biaffin GmbH (Kassel, Germany). Phosphatidylserine (PtdSer) was from Sigma–Aldrich (Poole, U.K.). Sheep anti-AMPKα1 and anti-AMPKα2 antibodies [29] and plasmids (pcDNA5/FRT) expressing FLAG-tagged AMPKα1, AMPKα1 Ser487Ala, AMPKα2 or AMPKα2 Ser491Ala [17] were a generous gift from Prof. D.G. Hardie (University of Dundee, U.K.). HeLa cells stably expressing wild-type LKB1 have been described elsewhere [30] and were kindly provided by Prof. D. Alessi (University of Dundee, U.K.). Plasmids (pBΔG) expressing full-length bovine PKCα, human PKCβ1 and human PKCβ2 have been described previously [31]. SV40-immortalised mouse embryonic fibroblasts (MEFs) lacking AMPKα1 and AMPKα2 have been described elsewhere [32] and were kindly provided by Dr. B. Viollet (Institut Cochin, Paris, France). All other reagents were from sources described previously [33–35].

### Cell culture

HAECs and HUVECs were grown in MV2 medium (Promocell, Heidelberg, Germany) and passaged when at 80% confluence. Cells were used for experiments between passages 3 and 6 as described previously [33–35]. For experiments in which extracellular Ca^2+^ was depleted, cells were incubated in KRH buffer [20 mmol/l HEPES–NaOH (pH 7.4), 119 mmol/l NaCl, 5 mmol/l NaHCO_3_, 5 mmol/l glucose, 4.8 mmol/l KCl, 1.2 mmol/l MgSO_4_, 1.2 mmol/l NaH_2_PO_4_, 2.5 mmol/l CaCl_2_] or KRH without CaCl_2_ supplemented with 1 mmol/l EGTA for 2 h prior to stimulation with VEGF or AICAR. HeLa cells and HEK293 cells were cultured in DMEM supplemented with 10% (v/v) foetal calf serum (FCS). HeLa cells stably expressing LKB1 and MEFs lacking AMPKα1 and AMPKα2 (AMPK KO MEFs) were cultured as described previously [32,35].

### Transient transfection of AMPK KO MEFs, HeLa cells or HEK293 cells

AMPK KO MEFs (∼60% confluence), HeLa cells or HEK293 cells (∼80% confluence) in six-well plates were incubated in 1 ml/well Opti-MEM. DNA–Lipofectamine 2000 complexes [150 µl of 13 µg/ml plasmid DNA, 2.5% (v/v) Lipofectamine 2000 for AMPK KO MEFs; 400 µl of 5 µg/ml plasmid DNA, 0.5% (v/v) Lipofectamine 2000 for HeLa cells; 150 µl of 13 µg/ml plasmid DNA, 3.3% (v/v) Lipofectamine 2000 for HEK293 cells] were added dropwise to each well. Cells were incubated at 37°C for 2 h (AMPK KO MEFs) or 4 h (HeLa and HEK cells) before the transfection media were replaced with 2 ml/well complete culture media. After incubation overnight, medium was replaced with serum-free DMEM for 2 h and cell lysates were prepared.

### siRNA-mediated down-regulation of PKC isoforms in HUVECs

Cells at ∼70% confluence in six-well plates were incubated in 750 µl/well Opti-MEM and 150 µl of 3.2 µmol/l siRNA complexed with 8% (v/v) HiPerFect in Opti-MEM were added dropwise to each well. Cells were incubated at 37°C for 3 h prior to the addition of 1.5 ml/well MV2 medium. After further incubation for 48 h at 37°C, medium was replaced with Medium 199 for 2 h prior to stimulation in the presence or absence of VEGF (10 ng/ml, 5 min).

### Preparation of cell lysates, SDS–PAGE and immunoblotting

Cell lysates were prepared; proteins were resolved by SDS–PAGE and subjected to immunoblotting with the antibodies indicated as described previously [33–35]. Proteins were visualised using infrared dye-labelled secondary antibodies on a LiCor Odyssey infrared imaging system and analysed using Image J software.

### Immunoprecipitation and assay of AMPK activity

Cell lysates (0.1 mg) were added to 1 μg of sheep anti-AMPKα1 or AMPKα2 antibodies bound to Protein G Sepharose (5 μl packed volume/immunoprecipitation) in IP buffer [50 mmol/l Tris–HCl (pH 7.4 at 4°C), 150 mmol/l NaCl, 50 mmol/l NaF, 5 mmol/l Na_4_P_2_O_7_, 1 mmol/l Na_3_VO_4_, 1 mmol/l EDTA, 1 mmol/l EGTA, 1 mmol/l DTT, 0.1 mmol/l benzamidine, 0.1 mmol/l phenylmethylsulphonyl fluoride, 5 μg/ml soybean trypsin inhibitor, 1% (v/v) Triton X-100, 1% (v/v) glycerol] and mixed for 3 h at 4°C. Immunodepleted lysates were collected and immunoprecipitates were washed with high-salt IP buffer (IP buffer containing 1 mol/l NaCl, 2 × 1 ml), IP buffer (2 × 1 ml) and 1 × 1 ml HBD buffer [50 mmol/l HEPES–NaOH (pH 7.4), 0.02% (v/v) Brij-35 and 1 mmol/l DTT]. Immunoprecipitates and immunodepleted lysates were subjected to SDS–PAGE and immunoblotting or assayed for AMPK activity using the SAMS substrate peptide as described previously [33].

### Immunoprecipitation and *in vitro* phosphorylation of AMPK

HEK293 cells were infected with adenoviruses expressing a myc-tagged kinase dead AMPKα1 [36] as described previously [33,35] or transiently transfected with FLAG-tagged AMPKα1, AMPKα1 Ser487Ala, AMPKα2 or AMPKα2 Ser491Ala and cell lysates were prepared. Kinase-dead AMPK was immunoprecipitated with agarose-conjugated mouse anti-myc tag antibodies. FLAG-tagged AMPK was immunoprecipitated with mouse anti-FLAG antibodies. Immunoprecipitates were incubated with 100 U/ml CIAP for 30 min at 30°C and washed three times each with IP buffer and HEPES–DTT buffer [50 mmol/l HEPES (pH 7.4) and 1 mmol/l DTT] prior to incubation in the presence or absence of 0.02 or 0.1 U of PKC (active rat brain) or Akt1 (human recombinant), 1.7 mmol/l Ca^2+^, 0.6 mg/ml PtdSer [prepared by sonication (1 h, 50°C) in HEPES–DTT buffer] and 0.2 mmol/l ATP, 6 mmol/l MgCl_2_ for 30 min at 30°C. AMPK immunoprecipitates were centrifuged and the resultant pellets were washed with IP buffer and HEPES–DTT buffer prior to SDS–PAGE and immunoblotting.

### Human muscle samples

Particulate membrane fractions from muscle (vastus lateralis) biopsy lysates were prepared in an earlier study [37], from volunteers of European descent in which the insulin sensitivity index (ISI) derived by Matsuda and DeFronzo [38] was also calculated. Fractions from six individuals were chosen for analysis due to their range of ISI and availability. Muscle biopsy lysates were obtained with informed consent from individuals with ethical approval for these analyses obtained from the National Research Ethics Service (Proportionate Review Sub-committee of the NRES Committee West Midlands — Solihull).

### Statistics

Statistically significant differences were determined using a two-tailed Student's *t*-test or ANOVA as appropriate, with *P *< 0.05 deemed significant.

## Results

### VEGF stimulates AMPKα1 Ser487 phosphorylation in human endothelial cells in a manner dependent on extracellular Ca^2+^ but independent of Akt or ERK1/2

**Figure 1.**
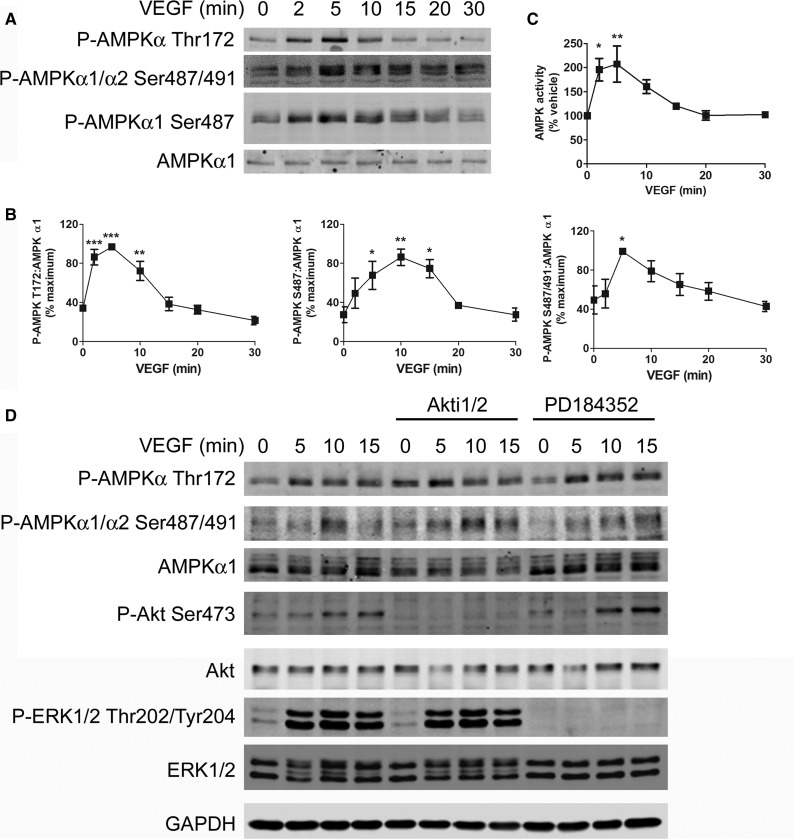
VEGF stimulates AMPK Ser487/491 phosphorylation independent of Akt or ERK1/2. HAECs were incubated (**A**–**C**) in 10 ng/ml VEGF for the times indicated and lysates were prepared. (**A** and **B**) Proteins were resolved by SDS–PAGE and immunoblotted with the antibodies indicated, or (**C**) AMPK was immunoprecipitated and activity was assayed. (**D**) HAECs were pre-incubated in the presence or absence of 1 µmol/l Akti1/2 or PD184352 for 60 min prior to VEGF stimulation (10 ng/ml, for the times indicated), lysates were prepared and proteins were resolved by SDS–PAGE and immunoblotted with the antibodies indicated. (**A** and **D**) Representative immunoblots are shown, repeated on two further occasions with similar results. (**B**) Densitometric quantification of immunoblots from 3 to 5 independent experiments (mean ± SEM). (**C**) AMPK activity (mean ± SEM). *P < 0.05, **P < 0.01, ***P < 0.005 relative to the absence of VEGF.

Stimulation of HAECs with 10 ng/ml VEGF rapidly stimulated phosphorylation of AMPKα Thr172, reaching a maximum after 5 min, before returning to basal levels by 20 min (Figure 1A,B). Both AMPKα1 and AMPKα2 isoforms are present in HAECs, but complexes containing AMPKα1 account for almost all of the basal and VEGF-stimulated AMPK activity in whole cell lysates (Supplementary Figure S1A,B). VEGF stimulated a significant increase in phosphorylation when using antibodies that recognise AMPKα1 Ser487 alone or both AMPKα1/α2 Ser487/491, which reached a maximum between 5 and 10 min before returning to basal levels by 30 min (Figure 1A,B). VEGF also stimulated AMPKα1 Ser487 in HUVECs with similar kinetics (Supplementary Figure S1C). VEGF rapidly stimulated AMPK activity within 2 min, reaching a maximum after 5 min before returning to basal levels (Figure 1C). To determine the kinase responsible for regulating VEGF-stimulated AMPKα Ser487/491 phosphorylation, selective kinase inhibitors were used. Pre-incubation of HAECs with the Akt inhibitor Akti-1/2 (Akt inhibitor VIII) or the MEK1/2 inhibitor PD184352 had no effect on VEGF-stimulated AMPKα Ser487/491 phosphorylation, despite completely inhibiting Akt Ser473 and ERK1/2 Thr202/Tyr204 phosphorylation, respectively (Figure 1D). Supporting the Akt-independent phosphorylation of AMPKα Ser487/491, stimulation of HAECs with insulin robustly stimulated Akt, but had no effect on AMPKα1 Ser487 phosphorylation (Supplementary Figure S1D). Furthermore, the phosphatidylinositide-3′-kinase (PI3K) inhibitor, wortmannin, completely inhibited VEGF-stimulated Akt Ser473 phosphorylation, without affecting VEGF-stimulated AMPKα1 Ser487 phosphorylation (Supplementary Figure S1E–G).

**Figure 2.**
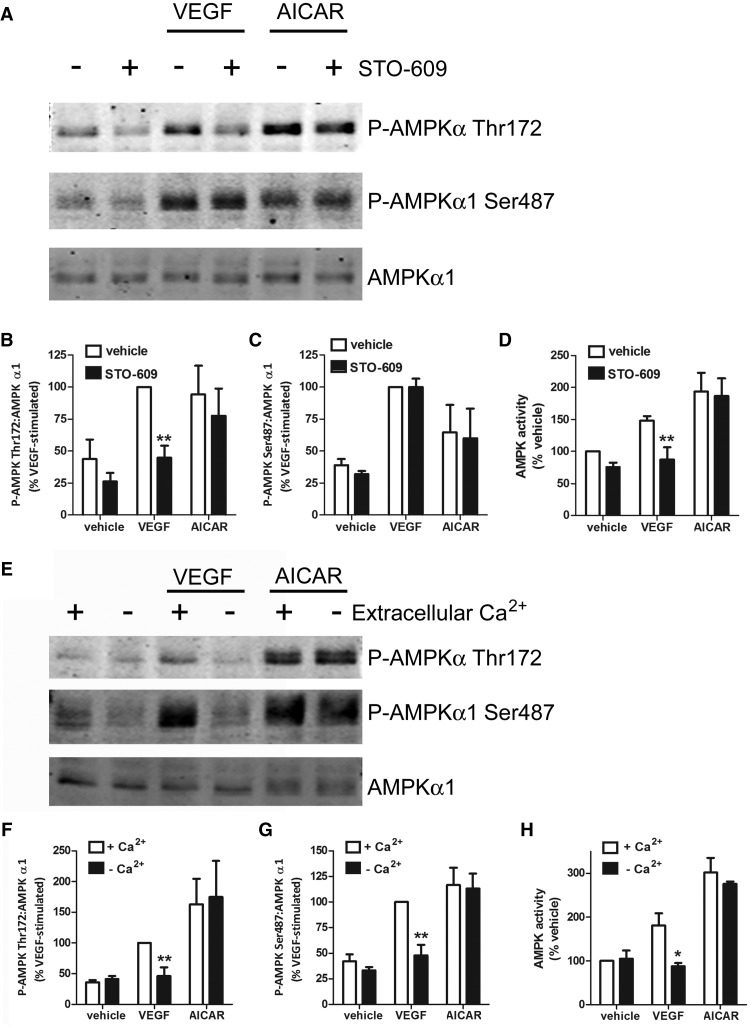
VEGF-stimulated AMPKα1 Ser487 phosphorylation is sensitive to Ca^2+^ removal but insensitive to CaMKK inhibition. HAECs were (**A**–**D**) incubated in the presence or absence of 10 µmol/l STO-609 for 60 min or (**E**–**H**) presence or absence of extracellular Ca^2+^ for 60 min, prior to stimulation with VEGF (10 ng/ml, 5 min) or AICAR (2 mmol/l, 45 min). HAEC lysates were prepared; (**A**–**C** and **E**–**G**) proteins were resolved by SDS–PAGE and immunoblotted with the antibodies indicated or (**D** and **H**) AMPK was assayed. (**A** and **E**) Representative immunoblots are shown, repeated with similar results on two further occasions. Densitometric quantification of (**B** and **F**) AMPK Thr172 and (**C** and **G**) AMPKα1 Ser487 phosphorylation (mean ± SEM) from three or four independent experiments. (**D** and **H**) AMPK activity (mean ± SEM) from three independent experiments. *P < 0.05, **P < 0.01 relative to the absence of STO-609 or the presence of extracellular Ca^2+^.

We have previously demonstrated that VEGF-stimulated Thr172 phosphorylation is mediated by CaMKK [28,33] and autophosphorylation of Ser487 by AMPK has been reported *in vitro* [13]. We therefore examined whether VEGF-stimulated Ser487 phosphorylation was sensitive to CaMKK inhibition. Pre-incubation of HAECs with the CaMKK inhibitor STO-609 significantly inhibited VEGF-stimulated AMPKα Thr172 phosphorylation, without influencing Thr172 phosphorylation stimulated by the CaMKK-independent AMPK activator, AICAR, demonstrating that STO-609 does not inhibit AMPK directly in these experiments (Figure 2A,B). In contrast, inhibition of CaMKK activity had no effect on VEGF-stimulated AMPKα1 Ser487 phosphorylation, arguing against VEGF-stimulated autophosphorylation of Ser487 (Figure 2A,C). Intriguingly, AICAR also stimulated AMPKα1 Ser487 phosphorylation in a CaMKK-independent manner in HAECs (Figure 2A,C). STO-609 inhibited VEGF-stimulated AMPK activity, but was without effect on AICAR-stimulated AMPK activity (Figure 2D). To determine whether increases in intracellular Ca^2+^ concentrations were important in VEGF-stimulated Ser487 phosphorylation, HAECs were incubated in the absence of extracellular Ca^2+^ and VEGF-stimulated Ser487 phosphorylation was assessed. Depletion of extracellular Ca^2+^ significantly inhibited VEGF-stimulated phosphorylation of AMPKα at Thr172 and Ser487, but had no effect on AICAR-stimulated phosphorylation of AMPK at these sites (Figure 2E–G). Depletion of Ca^2+^ inhibited VEGF-stimulated AMPK activity, but was without effect on AICAR-stimulated AMPK activity, in agreement with levels of Thr172 phosphorylation (Figure 2H).

### VEGF stimulates AMPKα1 Ser487 phosphorylation in a PKC-dependent manner

**Figure 3.**
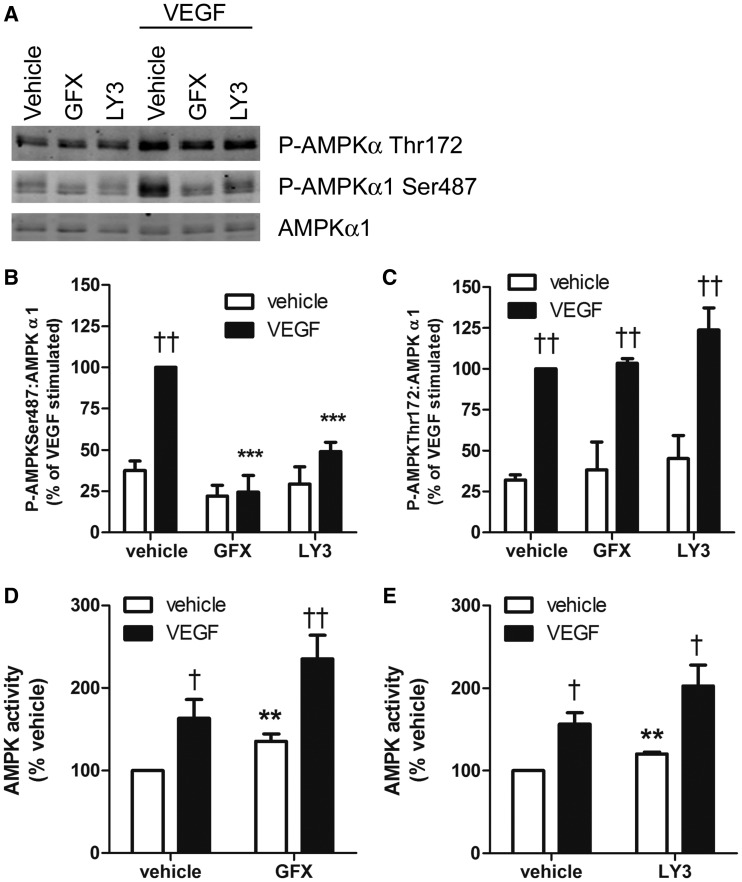
PKC inhibitors ablate VEGF-stimulated AMPKα1 Ser487 phosphorylation and stimulate AMPK activity. (**A**–**C**) HUVECs were pre-incubated in the presence or absence of either 1 μmol/l GF109203X (GFX) or LY333531 (LY3) for 1 h prior to stimulation with VEGF (10 ng/ml, 5 min). Cell lysates were prepared, proteins were resolved by SDS–PAGE and immunoblotted with the antibodies indicated. (**D** and **E**) HAECs were pre-incubated in the presence or absence of 1 μmol/l GFX or LY3 for 1 h prior to stimulation with VEGF (10 ng/ml, 10 min) and lysates were prepared. AMPK was immunoprecipitated from HAEC lystaes and assayed for AMPK activity. (**A**) Representative immunoblots are shown, repeated with similar results on two further occasions. (**B** and **C**) Densitometric quantification of immunoblots. Data are expressed as mean ± SEM relative to VEGF-treated HUVECs in the absence of inhibitor from three independent experiments. (**D** and **E**) Data represent mean ± SEM AMPK activity from eight and three independent experiments, respectively. ^†^*P* < 0.05, ^††^*P* < 0.01 relative to the absence of VEGF, **P < 0.01, ***P < 0.001 relative to the absence of PKC inhibitor.

VEGF stimulates numerous signalling pathways including the PKC family of kinases. As the conventional PKC isoforms (cPKC — α, β1/2 and γ) require Ca^2+^ for activation, we determined the effect of selective PKC inhibitors on VEGF-stimulated AMPK phosphorylation and activity. Pre-incubation of HUVECs with either GF109203X (cPKC-selective) or LY333531 (PKCβ-selective) completely inhibited VEGF-stimulated AMPKα1 Ser487 phosphorylation, without significantly altering VEGF-stimulated AMPKα Thr172 phosphorylation (Figure 3A–C). Furthermore, incubation of HAECs with either GF109203X or LY333531 significantly stimulated basal AMPK activity and tended to increase VEGF-stimulated AMPK activity (Figure 3D,E). To determine whether either PKC inhibitor directly influenced AMPK activity, immunoprecipitated AMPK was incubated with either GF109203X or LY333531 and AMPK activity was assessed. Neither GF109203X nor LY333531 had any significant direct effect on basal or AMP-stimulated AMPK activity *in vitro*, unlike the AMPK inhibitor, compound C (Supplementary Figure S2).

### PKC activators stimulate AMPKα1 Ser487 phosphorylation

**Figure 4.**
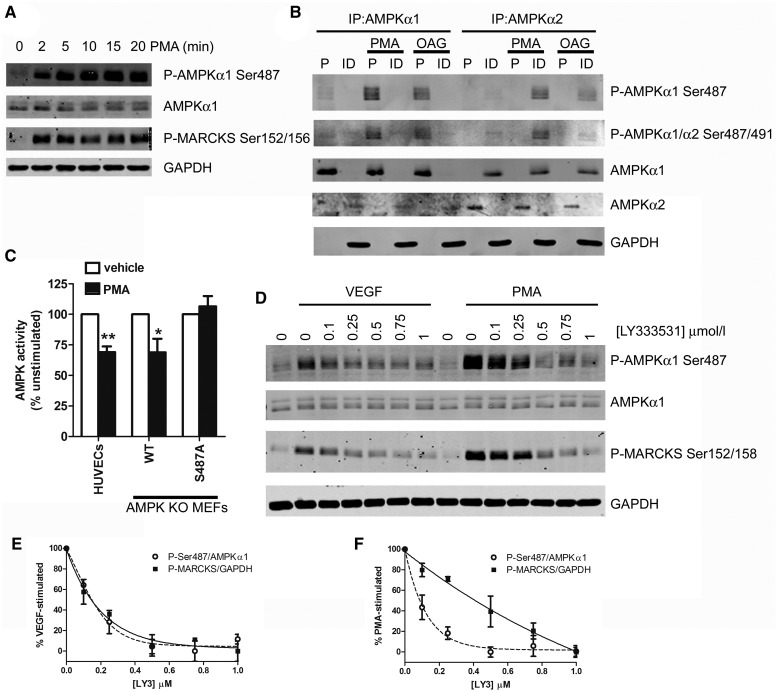
PKC activators stimulate AMPKα1 Ser487 phosphorylation. (**A**) HUVECs were stimulated with PMA (1 µmol/l) for the indicated durations and lysates prepared. Lysates were resolved by SDS–PAGE and immunoblotted using the antibodies indicated. (**B**) HUVECs were stimulated with PMA (1 μmol/l, 20 min) or OAG (0.1 mmol/l, 20 min), and lysates were prepared. AMPK complexes were immunoprecipitated with anti-AMPK α1 or α2 antibodies, and the pellets (P) and immunodepleted lysates (ID) were analysed by immunoblotting with the antibodies indicated. (**C**) AMPK KO MEFs were transiently transfected with AMPKα1 (wild type, WT) or AMPKα1 Ser487Ala. HUVECs or transfected MEFs were stimulated with PMA (1 μmol/l, 20 min) and lysates were prepared. AMPK activity was assessed in immunoprecipitates. (**D**) HUVECs were pre-treated with the indicated concentrations of LY333531 for 1 h prior to stimulation with VEGF (10 ng/ml, 5 min) or PMA (1 µmol/l, 20 min). Cell lysates were prepared and analysed by immunoblotting with the antibodies indicated. (**A**, **B** and **D**) Representative immunoblots are shown, repeated with similar results on two, one and two further occasions, respectively. (**C**) AMPK activity *P < 0.05, **P < 0.01 relative to the absence of PMA. (**E** and **F**) Densitometric quantification (mean ± SEM) of immunoblots in (**D**). Data are expressed relative to (**E**) VEGF-treated or (**F**) PMA-treated HUVECs in the absence of inhibitor from three independent experiments.

Supporting the potential role of PKC as an AMPKα1 Ser487 kinase, the synthetic PKC activator PMA rapidly stimulated phosphorylation of both the PKC substrate MARCKS and AMPKα1 Ser487 (Figure 4A). Furthermore, immunoprecipitation of AMPK complexes containing AMPKα1 or AMPKα2 from HUVECs stimulated with PMA or the diacylglycerol mimetic OAG demonstrated that both PMA and OAG stimulate AMPKα1 Ser487 phosphorylation (Figure 4B). In contrast, no immunoreactive bands were observed with an antibody that recognises both AMPKα1 Ser487 and AMPKα2 Ser491 in AMPKα2 immunoprecipitates or AMPKα1 immunodepleted lysates from PMA- or OAG-stimulated HUVECs, indicating that PKC activators stimulate AMPKα1 Ser487 and not AMPKα2 Ser491 phosphorylation (Figure 4B). In agreement with an inhibitory role of Ser487 phosphorylation, PMA inhibited AMPK activity in HUVECs by 31 ± 5% (Figure 4C). To determine whether the inhibition of AMPK activity by PMA was mediated by phosphorylation of Ser487, AMPKα KO MEFs were transfected with FLAG-tagged AMPKα1 or AMPKα1 in which Ser487 had been mutated to Ala and stimulated with PMA. PMA inhibited AMPK activity in cells expressing wild-type AMPKα1 by 31 ± 11%, yet had no effect in cells expressing AMPKα1 Ser487Ala (Figure 4C). Efficiency of transfection was similar for expression of FLAG-tagged wild-type or Ser487Ala mutant AMPKα1 (Supplementary Figure S3). To compare the sensitivity of VEGF- and PMA-stimulated AMPKα1 Ser487 phosphorylation with PKC inhibition, HUVECs were incubated with increasing concentrations of the PKCβ-selective inhibitor LY333531 prior to stimulation with VEGF or PMA. PMA (1 µmol/l) stimulated phosphorylation of both AMPKα1 Ser487 and the PKC substrate MARCKS to a greater extent than VEGF (10 ng/ml) (Figure 4D). The concentration dependence of LY333531-mediated inhibition of VEGF-stimulated phosphorylation of AMPKα1 Ser487 and MARCKS was almost identical, with an IC_50_ of ∼0.1–0.15 μmol/l (Figure 4E). In contrast, the concentration dependence of LY333531-mediated inhibition of PMA-stimulated phosphorylation of AMPKα1 Ser487 and MARCKS was different, whereby the IC_50_ for inhibition of AMPKα1 Ser487 was similar to VEGF-stimulated cells (∼0.1 μmol/l), but the IC_50_ for inhibition of MARCKS phosphorylation was greater (∼0.4 μmol/l; Figure 4F).

PMA-mediated phosphorylation of AMPKα1 Ser487 was not limited to endothelial cells, as PMA stimulated AMPKα1 Ser487 phosphorylation and MARCKS phosphorylation in HeLa cells, without significantly altering basal phosphorylation of the AMPK substrate, ACC. Pre-incubation with STO-609 reduced ACC phosphorylation in the presence or absence of PMA, but had no effect on PMA-stimulated AMPKα1 Ser487 or MARCKS phosphorylation (Supplementary Figure S4). In HeLa cells stably expressing LKB1, PMA stimulated AMPKα1 Ser487 phosphorylation and concomitantly inhibited basal and AICAR-stimulated ACC phosphorylation. AICAR had no effect on basal or PMA-stimulated AMPKα1 Ser487 phosphorylation in these cells (Supplementary Figure S4).

**Figure 5.**
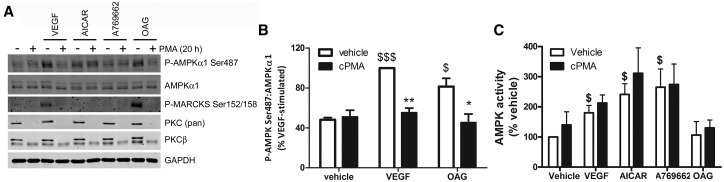
Down-regulation of PKC prevents VEGF-stimulated Ser487 phosphorylation. HUVECs were cultured for 20 h in the presence of 0.2 µmol/l PMA (cPMA) prior to stimulation with VEGF (10 ng/ml, 5 min), AICAR (2 mmol/l, 45 min), A769662 (100 µmol/l, 60 min) or OAG (100 µmol/l, 20 min). Cell lysates were prepared and (**A** and **B**) subjected to immunoblotting with the antibodies indicated or (**C**) AMPK activity was assayed. (**A**) Representative immunoblots are shown, repeated on two further occasions with similar results. (**B**) Densitometric quantification (mean ± SEM) of immunoblots from three independent experiments. (**C**) Data represent mean ± SEM AMPK activity from three independent experiments. **P < 0.01, *P < 0.05 relative to the absence of cPMA pre-treatment. ^$$$^*P* < 0.001, ^$^*P <* 0.05 relative to vehicle.

Chronic treatment of cells with PMA has been shown to down-regulate levels of PKC by stimulating its degradation [39]. To further investigate the role of PKC in the regulation of AMPKα1 Ser487 phosphorylation, HUVECs were incubated overnight with 200 nmol/l PMA prior to acute stimulation with VEGF, AICAR, the AMP-independent AMPK activator A769662 or OAG. Neither PKCβ nor immunoreactivity using a pan-specific anti-PKC antibody was detectable after chronic PMA treatment (Figure 5A). Chronic PMA treatment also down-regulated PKCα, PKCγ, PKCη, PKCθ and PKCμ in both HUVECs and HAECs (Supplementary Figure S5). PKCδ was unaffected by chronic PMA treatment, whereas PKCε, PKCζ and PKCλ were undetectable in HUVECs or HAECs (Supplementary Figure S5). Chronic PMA treatment completely inhibited the rapid phosphorylation of AMPKα1 Ser487 and MARCKS in response to VEGF or OAG (Figure 5A,B). AICAR did not stimulate MARCKS phosphorylation and AICAR-stimulated AMPKα1 Ser487 phosphorylation was unaffected by chronic PMA treatment (Figure 5A). The direct AMPK activator A769662 did not influence AMPKα1 Ser487 or MARCKS phosphorylation (Figure 5A). Despite reducing AMPKα1 Ser487 phosphorylation, chronic PMA treatment did not significantly alter AMPK activity under basal or VEGF/AICAR/A769662-stimulated conditions (Figure 5C).

### Up-regulation of PKC expression stimulates AMPKα1 Ser487 phosphorylation

**Figure 6.**
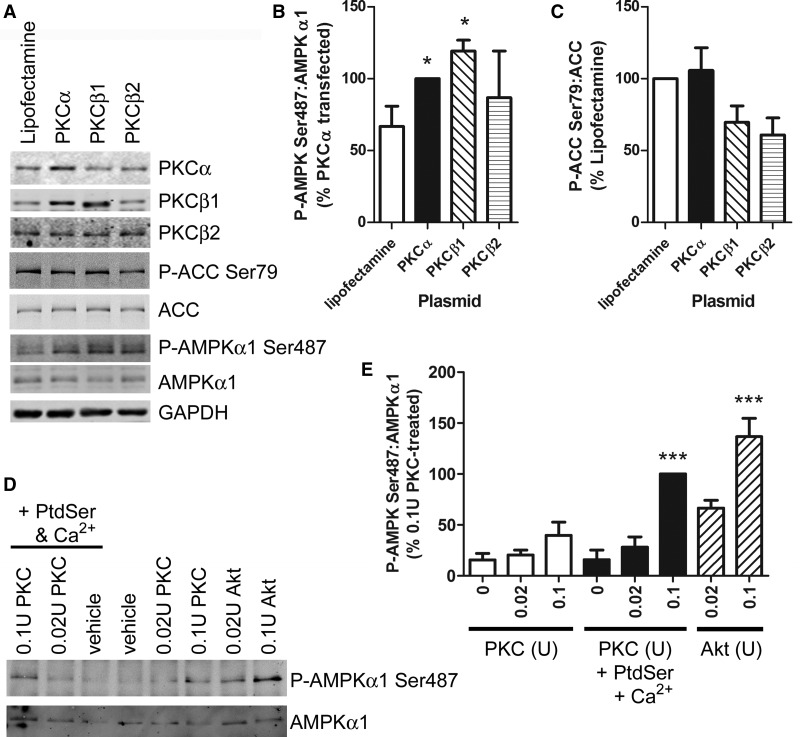
Overexpression of PKC increases basal Ser487 phosphorylation in HeLa cells and PKC is an *in vitro* AMPKα1 Ser487 kinase. (**A**–**C**) HeLa cells were transfected with vectors containing bovine PKCα, human PKCβ1 or human PKCβ2, and cell lysates were subjected to immunoblotting with the antibodies indicated. (**A**) Blots shown are representative of three independent experiments in each case. (**B** and **C**) Quantification (mean ± SEM) of AMPKα1 and ACC phosphorylation from three independent experiments. (**D** and **E**) Dephosphorylated, immunoprecipitated myc-tagged kinase-dead AMPKα1 was incubated in the presence of purified rat brain PKC or recombinant Akt1 (30 min, 30°C) as indicated in the presence or absence of Ca^2+^ and PtdSer. Proteins were resolved by SDS–PAGE and analysed by immunoblotting with the antibodies indicated. (**D**) Representative immunoblots from three independent experiments are shown. (**E**) Quantification (mean ± SEM) of P-Ser487 relative to AMPKα1 from three independent experiments. *P < 0.05, ***P < 0.001 relative to the absence of kinase.

Overexpression of bovine PKCα or human PKCβ1 in HeLa cells significantly increased AMPKα1 Ser487 phosphorylation. In contrast, overexpression of PKCβ2 tended to increase AMPKα1 Ser487 phosphorylation, yet this did not achieve statistical significance (Figure 6A,B). Overexpression of PKCβ1 and PKCβ2 tended to reduce basal ACC phosphorylation (Figure 6A,C). Furthermore, purified PKC phosphorylated kinase inactive AMPKα1 *in vitro* in the presence of PtdSer and Ca^2+^, with similar efficiency to a comparable activity of Akt (Figure 6D,E). PKC did not increase anti-phospho-AMPK Ser487 immunoreactivity of AMPKα1 in which Ser487 had been mutated to Ala *in vitro* (Supplementary Figure S6A). Specific siRNA-mediated down-regulation of PKCα also substantially down-regulated PKCβ1 levels, yet this was not associated with reduced VEGF-stimulated AMPKα1 Ser487 phosphorylation in HUVECs (Supplementary Figure S6B,C).

### Inhibition of PKCµ does not ablate VEGF-stimulated AMPKα1 Ser487 phosphorylation

**Figure 7.**
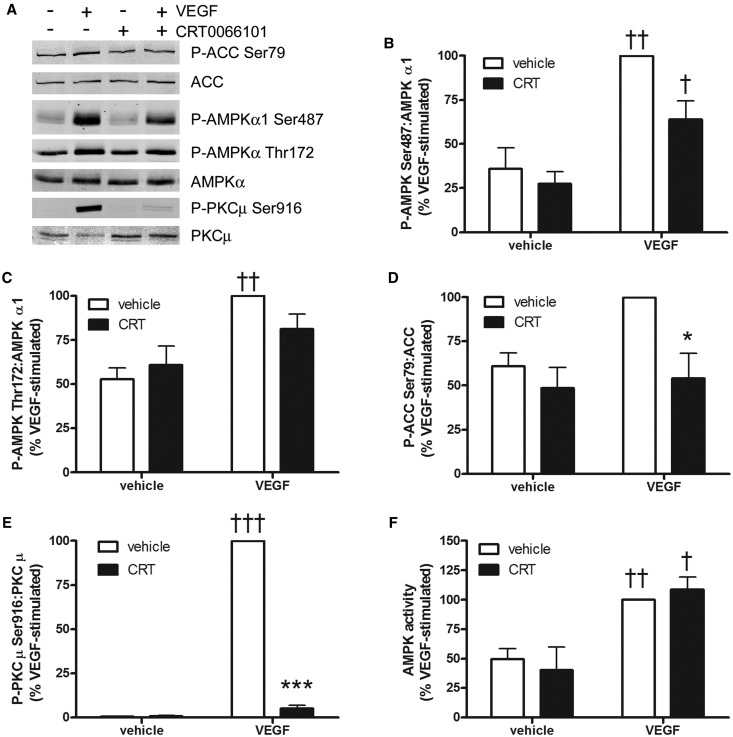
Role of PKCµ in VEGF-stimulated AMPKα1 Ser487 phosphorylation. HAECs were pre-incubated in the presence or absence of 10 μmol/l CRT0066101 (CRT) for 1 h prior to stimulation with VEGF (10 ng/ml, 5 min). Cell lysates were prepared, and (**A**–**E**) proteins were resolved by SDS–PAGE and immunoblotted with the antibodies indicated or (**F**) AMPK was assayed. (**A**) Representative immunoblots are shown, repeated with similar results on three further occasions. (**B**–**E**) Densitometric quantification of immunoblots. Data are expressed as mean ± SEM relative to VEGF-treated HAECs in the absence of inhibitor from four independent experiments. (**F**) AMPK activity (mean ± SEM) from three independent experiments. ^†^*P* < 0.05, ^††^*P* < 0.01, ^†††^*P* < 0.001 relative to the absence of VEGF, *P < 0.05, ***P < 0.001 relative to the absence of CRT.

While this manuscript was in preparation, it was reported that PKD1 (the mouse orthologue of human PKCμ) phosphorylates AMPKα2 Ser491 *in vitro* with similar efficiency to Akt and that PMA stimulated AMPKα2 Ser491 in a mouse muscle cell line in a PKD1-dependent manner [18]. VEGF markedly increased PKCµ Ser916 phosphorylation in HAECs, an autophosphorylation site that correlates with PKCµ activity [40], and this was entirely blocked by the PKCµ inhibitor CRT0066101 (Figure 7). Pre-incubation with CRT0066101 tended to inhibit VEGF-stimulated AMPKα1 Ser487 phosphorylation, although this effect did not achieve statistical significance and completely inhibited VEGF-stimulated ACC phosphorylation without inhibiting VEGF-stimulated AMPK activity (Figure 7). Specific siRNA-mediated down-regulation of PKCµ had no effect on VEGF-stimulated AMPKα1 Ser487 phosphorylation in HUVECs (Supplementary Figure S5C).

### AMPKα1 Ser487 phosphorylation is inversely associated with insulin sensitivity in human muscle

**Figure 8.**
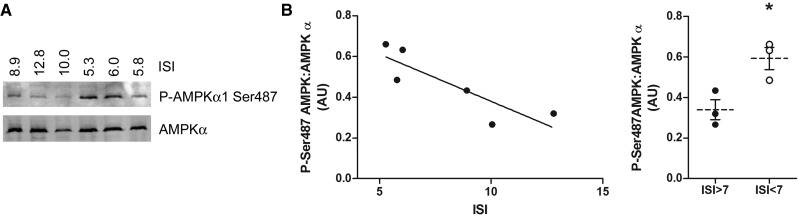
AMPKα1 Ser487 phosphorylation is inversely related to insulin sensitivity in human muscle. Human muscle biopsy membrane fractions were prepared in a previous study [35] and stored at −80°C. (**A**) Membrane fraction proteins of individuals of the indicated ISI were resolved by SDS–PAGE and immunoblotted using the antibodies shown. (**B**) Quantification (mean ± SEM) of AMPKα1 Ser487 phosphorylation relative to total AMPKα. *P < 0.05 comparing individuals with an ISI of <7 with those with an ISI of >7.

PKC isoform activation has been proposed to mediate lipid-induced insulin resistance in muscle, liver and vascular tissues [41,42]. We therefore assessed AMPKα1 Ser487 phosphorylation in muscle biopsy membrane fractions we obtained in a previous study from European men in which the ISI had been assessed [37]. Muscle microsomal AMPKα1 Ser487 phosphorylation showed a significant inverse association with ISI (*P* < 0.05, *r*^2^ = 0.7337; Figure 8). Furthermore, individuals with an ISI of <7 exhibited significantly higher levels of AMPKα1 Ser487 phosphorylation compared with those with an ISI of >7 (Figure 8). Phospho-MARCKS could not be detected in any muscle biopsy sample (data not shown).

## Discussion

The present study demonstrates that PKC isoforms stimulate AMPKα1 Ser487 phosphorylation, which is associated with reduced AMPK activity. Furthermore, phosphorylation of AMPKα1 Ser487 exhibits a strong inverse correlation with insulin sensitivity in human muscle. In addition, we demonstrate that an endogenous AMPK activator, VEGF, stimulates both Ser487 and Thr172 phosphorylation concomitantly via distinct signalling pathways in human endothelial cells.

Previous studies have demonstrated that Akt is an AMPKα1 Ser487 kinase that impairs activating Thr172 phosphorylation [13–15,17]. In contrast, this study demonstrates that VEGF-stimulated phosphorylation of AMPKα1 Ser487 is independent of Akt, using both Akti-1/2 and the PI3K inhibitor wortmannin. Furthermore, Akt activation by insulin is not associated with increased AMPKα1 Ser487 phosphorylation in HUVECs. These findings suggest that Akt activation alone is not sufficient to stimulate Ser487 phosphorylation in human endothelial cells, implying that Akt-mediated phosphorylation of this site is cell-specific and/or stimulus-specific. Furthermore, ERK1/2 activation does not underlie VEGF-stimulated AMPKα1 Ser487 phosphorylation as this was insensitive to an MEK1/2 inhibitor that ablated ERK1/2 phosphorylation.

AMPK autophosphorylation at Ser487/491 has been reported *in vitro* [13], yet STO-609 inhibited VEGF-stimulated AMPK activity without influencing Ser487 phosphorylation, indicating that AMPK autophosphorylation is unlikely to be the mechanism responsible. This is reinforced by the lack of effect of A769662 on endothelial cell AMPKα1 Ser487 phosphorylation, despite robustly activating AMPK. Intriguingly, AICAR stimulated AMPKα1 Ser487 phosphorylation in HAECs and HUVECs, as previously reported in neonatal rat cardiomyocytes, VSMCs and a mouse microglial cell line [24–26]. In contrast, AICAR had no effect in HeLa cells expressing LKB1, despite stimulating ACC phosphorylation. The mechanism by which AICAR stimulates Ser487 phosphorylation in human endothelial cells is distinct from the mechanism of VEGF, as it was unaffected by extracellular Ca^2+^ depletion or chronic PMA treatment. As AICAR stimulated AMPK activity in both human endothelial cells and HeLa cells expressing LKB1, yet only stimulated AMPKα1 Ser487 phosphorylation in endothelial cells, this also argues against autophosphorylation of AMPKα1 Ser487 being a major mechanism in human cells. We have previously reported that AICAR stimulates Akt phosphorylation and impairs ERK1/2 phosphorylation in HAECs [43], such that it remains possible that AICAR-stimulated Ser487 phosphorylation in human endothelial cells is mediated by Akt or an alternative AMPK-independent kinase.

The present study provides multiple lines of evidence that an isoform or isoforms of PKC phosphorylate AMPKα1 Ser487. Purified rat brain PKC phosphorylates AMPKα1 Ser487 *in vitro*, with a similar efficiency to the validated Ser487 kinase Akt. In addition, as the purified PKC is reported to consist primarily of PKCα, β and γ isoforms with lesser amounts of δ and ζ isoforms, this suggests that the conventional PKC isoforms are capable of phosphorylating AMPKα1 Ser487. Further evidence that conventional PKC isoforms phosphorylate AMPK is that overexpression of either PKCα or PKCβ1 or incubation with the DAG mimetic PMA each stimulated AMPKα1 Ser487 phosphorylation in HeLa cells. Similarly, both PMA and OAG stimulated AMPKα1 Ser487 phosphorylation in human endothelial cells. Furthermore, a physiological PKC activator, VEGF, stimulated AMPKα1 Ser487 phosphorylation in a manner sensitive to (i) two different PKC inhibitors and (ii) down-regulation of PKC by chronic PMA treatment. The sensitivity of VEGF-stimulated Ser487 phosphorylation to LY333531 was identical with that of VEGF-stimulated phosphorylation of the PKC substrate MARCKS, further providing evidence that Ser487 is a bona fide substrate for PKC or a PKC-activated protein kinase. Despite this, the sequence surrounding Ser487 in human AMPKα1 (PQRSGSVSNYRS) is not a conventional PKC consensus phosphorylation site, suggesting that it may be part of a non-contiguous consensus motif [44].

Others have recently reported that the murine PKCµ orthologue, PKD1, phosphorylates AMPKα2 *in vitro* and is responsible for PMA-stimulated AMPKα2 Ser491 phosphorylation in a mouse myotube cell line [18]. In the present study, PKCµ inhibition did not significantly attenuate VEGF-stimulated AMPKα1 Ser487 phosphorylation, indicating that PKCµ cannot be the principal VEGF-stimulated AMPKα1 Ser487 kinase. AMPKα2 is a minor catalytic isoform in human endothelial cells [43]. As neither PMA nor OAG stimulated detectable AMPKα2 Ser491 phosphorylation, this suggests that human AMPKα2 Ser491 is not a PKC substrate in endothelial cells and may be an autophosphorylation target, regulated independently of Ser487 as reported recently [17]. The different results reported in this and the study of Coughlan and co-workers [18] may reflect a species-specific role for PKCµ/PKD1. Indeed, the PKCµ inhibitor, CRT0066101, inhibited VEGF-stimulated ACC phosphorylation without affecting AMPK activity in immunoprecipitates, suggesting stimulation of PKCµ is not associated with AMPK inactivation in human endothelial cells. The reason for the lack of effect of CRT0066101 on AMPK activity while reducing VEGF-stimulated ACC phosphorylation is unclear. As the AMPK assay is performed in saturating levels of AMP, the inhibitory effect of CRT0066101 on ACC phosphorylation may reflect an inhibition of allosteric activation of AMPK or alternatively an off-target effect on ACC itself, increased ACC dephosphorylation or prevention of ACC phosphorylation by AMPK.

Phosphorylation of AMPKα1/α2 at Ser487/491 has been reported to inhibit AMPK activity [12–18]. This is supported by the present study, in which PMA stimulation of endothelial cells inhibited basal AMPK activity and attenuated AICAR-stimulated ACC phosphorylation in HeLa cells overexpressing LKB1. As the inhibition of AMPK activity by PMA was not observed in AMPK KO MEFs expressing mutant AMPKα1 Ser487Ala, it is highly likely that the inhibition of AMPK activity by PKC activators is due to Ser487 phosphorylation, although we cannot rule out that the effect of PMA is mediated by a Ser487 kinase other than PKC. Furthermore, incubation of HAECs with either of the two PKC inhibitors GF109203X or LY333531 stimulated basal AMPK activity. This is in agreement with a previous study, in which PMA decreased AMPK activity in a GF109203X-sensitive manner in rat cardiac myocytes [45]. Furthermore, GF109203X and another PKC inhibitor Ro-31–8425 have been reported to increase AMPKα Thr172 phosphorylation in mouse primary hepatocytes [46], and vinorelbine has been reported to stimulate PKC with concomitant inhibition of AMPK in HUVECs [47]. The present study extends these observations, demonstrating a clear inhibitory role for PKC isoforms in the regulation of AMPK inhibitory phosphorylation. In contrast with these and the present study, AMPK activation by preconditioning with ischaemia–reperfusion in rabbit myocardium has been reported to be attenuated by intravenous administration of GF109203X for 5 min [48], arguing that PKC inhibition is associated with reduced AMPK activity. Furthermore, PMA has been reported to rapidly stimulate AMPKα Thr172 phosphorylation in a GF109203X-dependent manner in THP-1 cells, such that PKC has been proposed to be upstream of LKB1 and AMPK [49]. The reasons for these discrepancies may be due to cell-specific actions of PKC or off-target effects of the reagents used, yet neither GF109203X nor LY333531 had any effect on immunoprecipitated human endothelial cell AMPK activity *in vitro*, suggesting that they do not directly stimulate AMPK.

Despite a weight of evidence supporting a role for PKC in directly phosphorylating AMPKα1 on Ser487, the nature of the PKC isoform responsible for VEGF-stimulated AMPKα1 Ser487 phosphorylation remains elusive. Although VEGF-stimulated AMPKα1 Ser487 phosphorylation was inhibited by the conventional (α, β1, β2 and γ) PKC isoform-selective inhibitor GF109203X, this may inhibit several other kinases in cell-free assays [50]. The PKCβ-selective inhibitor LY333531 has, however, been reported to exhibit much greater specificity [50] and did not influence AMPK activity directly in cell-free assays, but impaired VEGF-stimulated AMPKα1 Ser487 phosphorylation with identical kinetics to VEGF-stimulated MARCKS phosphorylation. Overexpression of PKCα or PKCβ1 was sufficient to cause a modest increase in AMPKα1 Ser487 phosphorylation in HeLa cells, yet down-regulation of PKCα (or PKCμ) had no effect on VEGF-stimulated AMPKα1 Ser487 phosphorylation in HUVECs. As VEGF-stimulated AMPKα1 Ser487 phosphorylation is mimicked by OAG and inhibited by LY333531 and chronic PMA incubation, it is likely that a DAG-stimulated PKC sensitive to both LY333531 and chronic PMA is responsible. It remains possible, therefore, that PKCγ, η or θ is responsible for the actions of VEGF, or that there is significant redundancy between PKC isoforms in their capacity to phosphorylate AMPKα1 Ser487.

PKC activation has been proposed to underlie lipid-induced insulin resistance in muscle, liver and vascular tissues [41,42], such that increased PKC-mediated inhibitory phosphorylation of AMPKα might explain reduced AMPK activity. In rodents, increased AMPKα1 Ser487 phosphorylation has been reported in brains and aortae from obese and diabetic mouse models, with concomitant increased basal phosphorylation of Akt [15,51], whereas increased AMPKα1/α2 Ser487/491 phosphorylation has been observed in muscles from glucose-infused rats [27]. In humans, infusion of glucose in healthy volunteers after sprinting increased phosphorylation of Ser487/491 in human vastus lateralis muscle, as assessed with an antibody that recognises both phosphorylated species [52]. We demonstrate a strong inverse relationship between insulin sensitivity and AMPKα1 Ser487 phosphorylation in vastus lateralis muscle from insulin-sensitive individuals. Ser487 phosphorylation in these muscle samples does not correlate with the phospho-Akt levels we have reported previously [37], such that it remains possible that PKC mediates Ser487 phosphorylation, although we were unable to detect any phospho-MARCKS as a surrogate of PKC activity. AMPKα1 is the minor catalytic subunit isoform in murine muscle, yet complexes containing AMPKα1 have been reported to contribute ∼50% of the basal AMPK activity in human vastus lateralis muscle [53], suggesting that increased Ser487 phosphorylation may markedly influence human muscle AMPK activity. We cannot, however, exclude the possibility that the AMPKα1 Ser487 phosphorylation observed in the present study is associated with vascular cells or leukocytes within the muscle.

Taken together, these data indicate that PKC is an AMPKα1 Ser487 kinase, which reduces AMPK activity. As PKC can be activated by lipid metabolites formed in metabolic tissues as a consequence of overnutrition, it can be speculated that PKC-mediated phosphorylation of AMPKα1 Ser487 underlies the reduced AMPK activity observed in tissues from mouse models of overnutrition and insulin-resistant people. It remains to be characterised whether the functional consequences of Ser487-mediated AMPK inactivation contribute to the pathogenesis of insulin resistance, dysfunctional metabolism and their associated cardiovascular complications.

## Abbreviations

ACC: acetyl-CoA carboxylase
AICAR: 5-aminoimidazole-4-carboxamide ribonucleoside
Akt: protein kinase B
AMPK: AMP-activated protein kinase
CIAP: calf intestine alkaline phosphatase
DAG: diacylglycerol
ERK: extracellular signal-regulated kinase
GAPDH: glyceraldehyde 3-phosphate dehydrogenase
HAECs: human aortic endothelial cells
HUVECs: human umbilical vein endothelial cells
ISI: insulin sensitivity index
LKB1: liver kinase B1
MARCKS: myristoylated alanine-rich protein kinase C substrate
MEF: mouse embryonic fibroblast
MEK1/2: mitogen-activated protein kinase kinase
OAG: 1-oleoyl-2-acetyl-*sn*-glycerol
PKA: cAMP-dependent protein kinase
PKC: protein kinase C
PMA: phorbol 12-myristate 13-acetate
VEGF: vascular endothelial growth factor.
